# Antifungal Susceptibility Pattern of *Candida glabrata* from a Referral Center and Reference Laboratory: 2012–2022

**DOI:** 10.3390/jof9080821

**Published:** 2023-08-03

**Authors:** Supavit Chesdachai, Zachary A. Yetmar, Nischal Ranganath, Jenna J. Everson, Nancy L. Wengenack, Omar M. Abu Saleh

**Affiliations:** 1 Division of Public Health, Infectious Diseases and Occupational Medicine, Department of Medicine, Mayo Clinic, Rochester, MN 55905, USA; yetmar.zachary@mayo.edu (Z.A.Y.); ranganath.nischal@mayo.edu (N.R.); abusaleh.omar@mayo.edu (O.M.A.S.); 2 Division of Clinical Microbiology, Department of Laboratory Medicine and Pathology, Mayo Clinic, Rochester, MN 55905, USA; everson.jenna@mayo.edu (J.J.E.); wengenack.nancy@mayo.edu (N.L.W.)

**Keywords:** *Candida glabrata*, epidemiological cutoff value, minimum inhibitory concentration, resistance, *Nakaseomyces glabrata*

## Abstract

The prevalence of invasive candidiasis caused by non-*Candida albicans* has rapidly increased. *Candida glabrata* (*Nakaseomyces glabrata*) is an important pathogen associated with substantial mortality. Our study examined the antifungal temporal susceptibility of *C. glabrata* and cross-resistance/non-wild-type patterns with other azoles and echinocandins. Laboratory data of all adult patients with *C. glabrata* isolated from clinical specimens at the Mayo Clinic, Rochester, from 2012 to 2022 were collected. Clinical and Laboratory Standards Institute (CLSI) and European Committee on Antimicrobial Susceptibility Testing (EUCAST) breakpoints were used. We obtained 1046 *C. glabrata* isolates from 877 patients. Using CLSI and EUCAST breakpoints, 187 (17.9%) isolates and 256 (24.5%) isolates were fluconazole-resistant, respectively. Focusing on *C. glabrata* bloodstream infections, fluconazole-resistance ranged from 16 to 22%. Among those 187 fluconazole-resistant isolates, 187 (100%) and 184 (98.4%) isolates were also voriconazole and posaconazole non-wild-type, respectively, with 97 (51.9%) isolates deemed non-wild type for itraconazole. The fluconazole susceptibility pattern has not changed over the past decade. The proportion of fluconazole-resistant *C. glabrata* is relatively high, which could be due to the complexity of patients and fluconazole exposure. Itraconazole appears to be a compelling step-down therapy for fluconazole-resistant *C. glabrata*, given the high proportion of wild-type isolates. Further research to examine clinical outcomes is warranted.

## 1. Introduction

*Candida* species are typical human microbiota that can be found on the skin and gastrointestinal mucosal surface of healthy individuals [1]. Invasive candidiasis is a deep-seated organ infection with *Candida* species that can occur with or without bloodstream infection or candidemia [2]. Invasive candidiasis leads to significant morbidity and mortality worldwide, especially among critically ill and immunocompromised patients. The estimated mortality of invasive candidiasis ranged from 20 to 40%, with a significant healthcare burden [3,4,5]. Important risk factors for invasive candidiasis include broad-spectrum antibiotic exposure, the colonization of *Candida*, the presence of a central venous catheter, and total parenteral nutrition [6]. 

*Candida glabrata*, which has an alternate taxonomic name of *Nakaseomyces glabrata* [7], is the second most common cause of invasive candidiasis, following *Candida albicans*. The prevalence of invasive *C. glabrata* is increasing, especially in North America [8,9]. In 2022, the World Health Organization issued the fungal priority pathogens list to guide research, development, and public health action. This list categorized *C. glabrata* into the high-priority group for two main reasons: First, it is the most common cause of non-*C. albicans* invasive candidiasis, which is associated with a high mortality, especially in those with hematologic malignancy, prior fluconazole exposure, or neutropenia. Second, data for resistance patterns, pathogenicity, and preventative measures are lacking [10,11]. 

The clinical management of invasive candidiasis from *C. glabrata* is challenging. It is well-established that *C. glabrata* has reduced susceptibility to several antifungal agents, and its susceptibility pattern has changed over time [12]. The first-line empiric therapy for invasive *C. glabrata* infection is an echinocandin, with high-dose fluconazole or voriconazole used as step-down options [13]. However, previous studies have demonstrated that the prevalence of fluconazole-resistant *C. glabrata* has gradually increased in the United States [9,14]. Additionally, some fluconazole-resistant *C. glabrata* isolates were resistant to voriconazole; however, the pattern of cross-resistance for echinocandin and non-wild type for itraconazole and posaconazole were not well-delineated, partly due to a lack of established breakpoints and differences in antifungal susceptibility techniques [15]. Given the geographic and temporal variability in the *C. glabrata* susceptibility profile, this current study aimed to describe the trend in the susceptibility pattern of *C. glabrata* and the rate of cross-resistance/non-wild type among the fluconazole-resistant isolates at our institution over the past decade. 

## 2. Materials and Methods

### 2.1. Data Collection and Objectives 

A retrospective review of laboratory data was conducted. All adult patients who received care at the Mayo Clinic, Rochester, Minnesota, USA, with *C. glabrata* isolated from clinical specimens from 1 January 2012 to 31 May 2022 were included in the study. The patients who had more than one isolate of *C. glabrata* from an anatomically contiguous site were counted as the same isolate. The patients who had more than one isolate from the same anatomical site were considered a different isolate if the subsequent cultures were obtained more than two weeks from when the first isolate was. Baseline demographic data, including age, sex, and race, were obtained from Mayo Clinic Data Explorer software, which provided demographic data from the Mayo Clinic’s electronic medical records. This study was approved by the Mayo Clinic Institutional Review Board (study number 22-008030).

The primary objective of the study was to examine the antifungal susceptibility pattern of *C. glabrata* via the use of Clinical and Laboratory Standards Institute (CLSI) and European Committee on Antimicrobial Susceptibility Testing (EUCAST) breakpoints. The secondary objectives were (1) to determine the prevalence of fluconazole-resistant isolates over time and (2) to determine the prevalence of cross-resistance/non-wild type of fluconazole-resistant isolates to other antifungals. Cross-resistance was defined as fluconazole-resistant *C. glabrata* isolates with a minimal inhibitory concentration (MIC) above the breakpoint of echinocandins. Non-wild type was defined as fluconazole-resistant *C. glabrata* isolates with a minimal inhibitory concentration (MIC) above the epidemiological cut-off value (ECV or ECOFF) of other azoles.

### 2.2. C. glabrata Identification and Antifungal Susceptibility Testing

Blood was collected and inoculated into Becton Dickinson BD BACTEC™ blood culture media bottles (Becton, Dickinson, and Company, Franklin Lakes, NJ, USA). Blood culture bottles were incubated at 35 °C in the BD BACTEC™ FX blood culture system for five days. Blood culture bottles that showed yeast from the Gram stain were subcultured into a Sabouraud Dextrose Agar plate at 28–30 °C in room air and examined daily for growth. The pure growth of yeast was further identified via matrix-assisted laser desorption/ionization-time of flight mass spectrometry (MALDI-TOF MS) using a Bruker Daltonics MALDI Biotyper^®^ System (Bruker Daltonics, Bremen, Germany). Clinical specimens other than blood were cultured to agar plates including inhibitory mold agar and brain heart infusion agar containing chloramphenicol, gentamycin, and, for respiratory specimen sources, cycloheximide. Plates were incubated at 30 ± 1 °C in ambient air for up to 28 days. Isolates were identified as *C. glabrata* using either MALDI-TOF MS or the D2 region of the large subunit ribosomal ribonucleic acid (D2 rRNA) gene sequencing, as previously described [16,17]. Two cryptic species, including *C. nivariensis* or *C. bracarensis*, were not included in this study.

After *C. glabrata* was identified, an antifungal susceptibility test was performed using a Sensitre Y09 microtitre plate (Thermo Fisher Scientific, Waltham, MA, USA) following CLSI guidelines and M27 guidelines [18]. *C. glabrata* clinical breakpoints and ECVs/ECOFFs were based on (1) CLSI documents M27M44S and M57S [18,19] as well as (2) breakpoint tables for the interpretation of MICs for antifungal agents version 10.0 from the EUCAST [20]. 

### 2.3. Statistical Analysis

Descriptive statistics were used, including the median and interquartile range (IQR) for continuous variables in addition to frequency and percentage for categorical variables. All analyses were performed using BlueSky Statistics version 7.40 (BlueSky Statistics LLC, Chicago, IL, USA). 

## 3. Results

### 3.1. Isolate Description

A total of 1046 non-duplicated *C. glabrata* isolates were obtained from 877 patients during the study period. The median age was 66 (IQR: 54–75) years, with 466 (53.1%) patients being female and 776 (88.5%) being white. The most common specimen source was intra-abdominal fluid (number (n) = 329, 31.5%), followed by blood (n = 247, 23.6%), urine (n = 196, 18.7%), skin/mucosal swab (n = 127, 12.1%), bone and joint tissue/fluid (n = 81, 7.7%), respiratory specimens (n = 49, 4.7%), and tissue from the mediastinum and vessels (n = 17, 1.6%). The summary of baseline characteristics can be found in Table 1. 

### 3.2. Susceptibility Pattern

**Figure 1:**
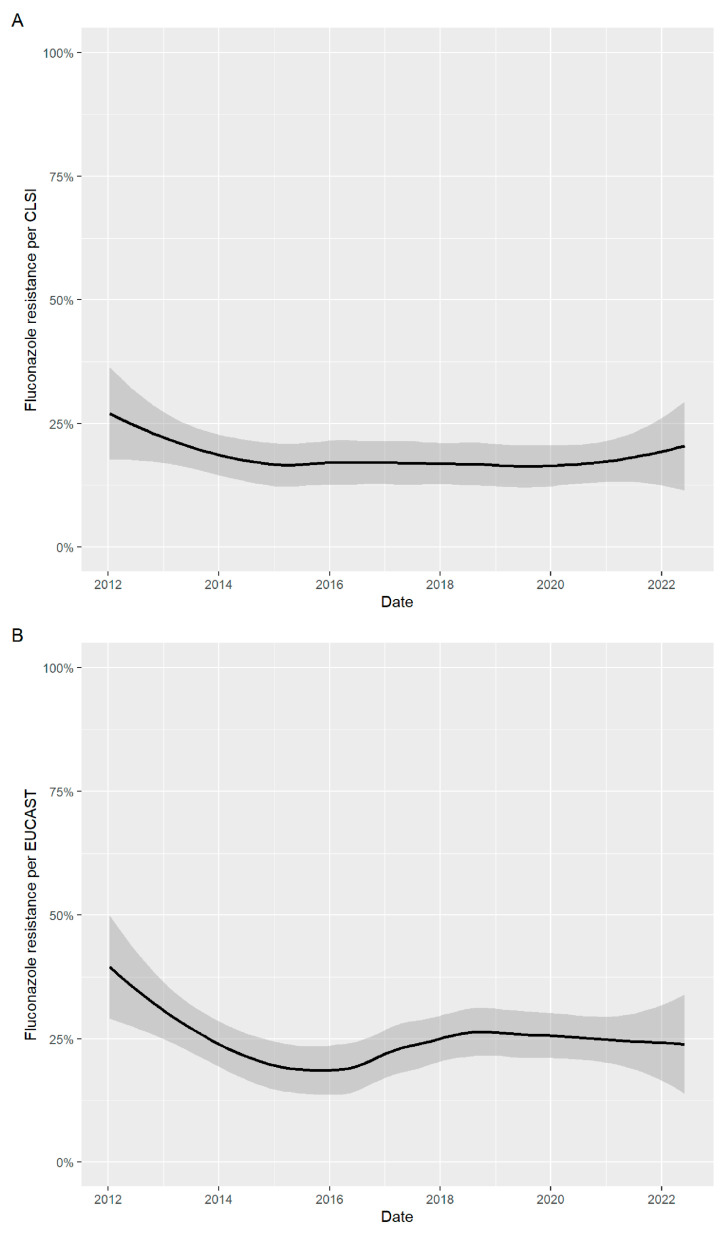
The proportion of fluconazole-resistant isolates was not different between years. A: based on CLSI breakpoint and B: based on EUCAST breakpoint.

The in vitro susceptibility profile of all *C. glabrata* isolates is provided in Table 2. One hundred and eighty-seven (17.9%) isolates and two hundred and fifty-six (24.5%) isolates were fluconazole-resistant based on CLSI and EUCAST breakpoints, respectively. The highest proportion of fluconazole-resistant isolates was obtained from skin/mucosal swabs (29.1% for CLSI and 38.6% for EUCAST breakpoints). For blood isolates, fluconazole-resistant *C. glabrata* was found in 40 (16.2%) and 53 (21.5%) isolates based on CLSI and EUCAST breakpoints, respectively. Fluconazole susceptibility by specimen source is provided in Table 3. The proportion of fluconazole-resistant isolates based on CLSI and EUCAST breakpoints was similar over the 10-year study period (Figure 1). 

For other azoles without an established susceptibility breakpoint, the number of non-wild-type isolates was 100 (9.6%, CLSI) and 106 (10.1%, EUCAST) for itraconazole; 455 (43.5%, CLSI) and 146 (14.0%, EUCAST) for voriconazole; and 296 (28.3%, CLSI) and 296 (28.3%, EUCAST) for posaconazole. For caspofungin and micafungin, the proportion of resistant isolates ranged from 1.9 to 3.8%. The number of anidulafungin-resistant isolates was 21 (2.0%) for the CLSI and 124 (11.9%) for the EUCAST due to different breakpoints. No isolates were non-wild-type amphotericin based on the CLSI ECV, and only 13 (1.2%) isolates were amphotericin-resistant based on the EUCAST breakpoint. 

Figure 1: Fluconazole susceptibility pattern based on year.

### 3.3. Cross-Resistance/Non-Wild-Type Pattern of Fluconazole-Resistant Isolates

Based on the CLSI ECV, 187 (100%) and 184 (98.4%) fluconazole-resistant isolates were voriconazole and posaconazole non-wild-type, respectively; however, the number of itraconazole non-wild-type isolates was only 97 (51.9%). For echinocandins, 10 (5.3%) isolates had caspofungin cross-resistance, and 8 (4.3%) isolates were micafungin and anidulafungin cross-resistant. There was no amphotericin non-wild-type isolate among the fluconazole-resistant isolates. Cross-resistance/non-wild type based on the EUCAST breakpoint can be found in Appendix Table A1.

Among 40 fluconazole-resistant blood culture isolates with CLSI ECVs, the highest proportion of non-wild-type isolates was posaconazole (n = 39, 97.5%), followed by voriconazole (n = 34, 85.0%) and itraconazole (n = 22, 55.0%). The rate of cross-resistance to echinocandins was 5.0% for caspofungin (n = 2) as well as anidulafungin (n = 2), and 2.5% for micafungin (n = 1). Cross-resistance/non-wild-type based on the EUCAST breakpoint for blood isolates can be found in Appendix Table A2.

## 4. Discussion

Our study examined 1046 isolates of *C. glabrata* over the past decade. The proportion of fluconazole-resistant isolates was 18% and 25% based on the CLSI and EUCAST breakpoints, respectively. The highest proportion of fluconazole-resistant isolates was from the skin and mucosa, although the clinical significance of susceptibility testing in this setting remains unclear. There was no appreciable change in the prevalence of fluconazole-resistant *C. glabrata* during the past ten years. Additionally, the overall rate of echinocandin and amphotericin resistance/non-wild-type isolates remains low. Focusing on *C. glabrata* bloodstream infections, fluconazole resistance ranged from 16% to 22%, significantly higher than previously reported. Pfaller et al. reported the first multicenter *Candida* bloodstream infection surveillance from 1992 to 2001 after the introduction of fluconazole in 1990, with a *C. glabrata* fluconazole resistance rate of approximately 9% [21,22]. A recent study updated the susceptibility pattern of 2860 *C. glabrata* isolates worldwide from 1997 to 2016 via the use of the SENTRY Antifungal Surveillance Program. The proportion of fluconazole-resistant *C. glabrata* increased from 8.6% in 1997 to 10.1% in 2014. The highest rate of fluconazoleresistance in North America was 11% [14]. Previous studies have postulated that the increasing trend towards fluconazole-resistant *C. glabrata* may be due to a rise in patient complexity, prior fluconazole exposure, and aging populations [23,24]. Additionally, owing to selection pressure, prior fluconazole exposure increased the risk of invasive non-*C. albicans* infections, especially in patients with hematologic malignancies, bone marrow transplants, and critical illnesses [25,26,27]. Although detailed clinical characteristics were not examined in our study, we speculate that these risk factors likely remain applicable among our complex patient population. 

The variability in the CLSI and EUCAST breakpoints for *C. glabrata* is notable. Both CLSI and EUCAST use a broth microdilution technique to obtain MIC data. Although some processes were different between the CLSI and EUCAST, such as inoculum density, incubation time, type of well, glucose content, and endpoint, the breakpoints for fluconazole are comparable between the two standard-setting organizations [28]. The clinical breakpoint was established by taking the clinical outcome and pharmacology of the drug into account. There is no established CLSI and EUCAST clinical breakpoint of *C. glabrata* for itraconazole, voriconazole, and posaconazole. Therefore, the ECV (for the CLSI) and ECOFF (for the EUCAST) are utilized. The CLSI and EUCAST cutoffs for posaconazole are the same, while itraconazole has a one-dilution difference and voriconazole has a two-dilution difference. This difference in the cutoff does not impact the proportion of non-wild-type isolates for itraconazole and posaconazole, yet it made the interpretation of voriconazole non-wild-type challenging with 43.5% based on the CLSI ECV versus 14.0% based on the EUCAST ECOFF. It would be interesting to examine further how this difference affects the clinical decision on using voriconazole when considering both the ECV and ECOFF. 

There are numerous proposed resistance mechanisms for fluconazole with *C. glabrata* [29]. The primary mechanism is a “gain of function” of several genes, such as *C. glabrata* CDR1, CDR2, and SNQ2. Increased activity of these genes leads to an overexpression of the fluconazole binding target and a decrease in intracellular fluconazole concentration due to efflux pump enhancement [30,31,32]. Both in vitro and clinical studies have shown that *C. glabrata* also demonstrated a significant cross-resistance to other azoles [33,34]. Our study demonstrated that almost 100% of fluconazole-resistant isolates were voriconazole and posaconazole non-wild-type based on the CLSI breakpoint. Interestingly, only half of fluconazole-resistant isolates were itraconazole non-wild-type. A previous study from Castanheira et al. noted a similar observation [35]. The explanation for why itraconazole preserved the highest rate of wild-type isolates among other azoles remains unclear. The cross-resistance to echinocandins and amphotericin among fluconazole-resistant isolates was low, at < 10%, except for anidulafungin, which has a lower EUCAST breakpoint compared with the CLSI breakpoint. This observation is essential in clinical practice, primarily in the setting of highly fluconazole-resistant *C. glabrata*. Echinocandins are effective drugs against fluconazole-resistant *C. glabrata*, and remain a mainstay of therapy [13]. However, intravenous administration sometimes creates logistical challenges. Itraconazole may be a compelling option for the step-down strategy, given its lower probability of being a non-wild type. Nevertheless, more clinical data on itraconazole use in this scenario are needed. 

Our study has several limitations. First, *C. glabrata* MIC data were obtained from laboratory data without a clinical course nor antifungal treatment correlation. Thus, it is impossible to examine the association between fluconazole resistance and clinical outcomes. Second, some isolates were obtained from the same patient within a different timeframe. Hence, some might develop emerging resistance from antifungal exposure related to the first episode, yet we could not clearly demonstrate a pattern in our study. Third, the MIC breakpoint for the CLSI and EUCAST have changed over time. However, we applied the current breakpoint to all of the isolates; therefore, it would not affect the trend of a resistance pattern. Additionally, although there are some differences between the CLSI and EUCAST methods, a previous study has shown that the MICs/ECVs/ECOFFs are comparable [36]. Fourth, multilocus genome sequencing was not performed in the resistant isolates to determine the resistance mechanism. Fifth, our hospital is a tertiary referral center, and these results may not be generalizable to other patient populations. Lastly, selection bias may especially occur with the isolates from a non-blood source. For example, one may request the identification of species level and susceptibility when the patient did not have a good clinical response. Otherwise, the isolate may not be identified at the species level. 

## 5. Conclusions

In conclusion, our study demonstrated a relatively high proportion of fluconazole-resistant *C. glabrata*, including 16–22% of blood isolates. Non-wild-type voriconazole and posaconazole were also noted, but the in vitro activity of itraconazole seems to be preserved. Further studies focusing on the clinical correlation and utility of itraconazole as a step-down therapy in fluconazole-resistant *C. glabrata* is warranted. 

## Figures and Tables

**Table 1:** Baseline characteristics.

	N	n (%) or Median (IQR)
**Female**	877	466 (53.1%)
**Median age (years)**	877	66 (IQR: 54–75)
**Race** - White - African American - Asian - American Indian/Alaska Native - Unknown/other	877	776 (88.5%) 29 (3.3%) 18 (2.1%) 10 (1.1%) 44 (5.0%)
**Source** - Intra-abdominal - Blood - Urinary - Skin and mucosal - Others *	1046	329 (31.5%) 247 (23.6%) 196 (18.7%) 127 (12.1%) 147 (14.1%)
**Isolates by Year of Recovery** - 2012 - 2013 - 2014 - 2015 - 2016 - 2017 - 2018 - 2019 - 2020 - 2021 - 2022	1046	87 (8.3%) 88 (8.4%) 94 (9.0%) 106 (10.1%) 91 (8.7%) 120 (11.5%) 110 (10.5%) 123 (11.8%) 81 (7.7%) 107 (10.2%) 39 (3.7%)

* Other sources included bone and joint tissue/fluid (81), respiratory specimens (49), and tissue from the mediastinum and vessels (17). Abbreviations: IQR = interquartile range; n = number.

**Table 2:** Antifungal susceptibility pattern of 1046 *Candida glabrata* isolates.

Drug	MIC (mg/L)											
	≤0.12	0.5	1	2	4	8	16	32	64	128	256	>256
Fluconazole, n (%)	1 (0.1)	1 (0.1)	10 (1.0)	35 (3.3)	118 (11.3)	311 (29.7)	314 (30.0)	69 (6.6)	44 (4.2)	68 (6.5)	47 (4.5)	28 (2.7)
SDD vs. R (CLSI)	859 (82.1%)								187 (17.9%)			
I vs. R (EUCAST)	790 (75.5%)							256 (24.5%)				
**Drug**	**MIC (mg/L)**											
	**≤0.015**	**0.03**	**0.06**	**0.12**	**0.25**	**0.5**	**1**	**2**	**4**	**8**	**16**	**>16**
Itraconazole, n (%)	1 (0.1)	4 (0.4)	8 (0.8)	29 (2.8)	131 (12.5)	471 (45.0)	254 (24.3)	42 (4.0)	6 (0.6)	1 (0.1)	8 (0.8)	91 (8.7)
WT vs. non-WT (CLSI)	946 (90.4%)									100 (9.6%)		
WT vs. non-WT (EUCAST)	940 (89.9%)								106 (10.1%)			
**Drug**	**MIC (mg/L)**											
	**≤0.008**	**0.015**	**0.03**	**0.06**	**0.12**	**0.25**	**0.5**	**1**	**2**	**4**	**8**	**>8**
Voriconazole, n (%)	1 (0.1)	4 (0.4)	14 (1.3)	39 (3.7)	174 (16.6)	359 (34.3)	237 (22.7)	72 (6.9)	62 (5.9)	59 (5.6)	19 (1.8)	6 (0.6)
WT vs. non-WT (CLSI)	591 (56.5%)						455 (43.5%)					
WT vs. non-WT (EUCAST)	900 (86.0%)								146 (14.0%)			
**Drug**	**MIC (mg/L)**											
	**≤0.008**	**0.015**	**0.03**	**0.06**	**0.12**	**0.25**	**0.5**	**1**	**2**	**4**	**8**	**>8**
Posaconazole, n (%)	1 (0.1)	0 (0)	3 (0.3)	10 (1.0)	8 (0.8)	41 (3.9)	182 (17.4)	505 (48.3)	161 (15.4)	1 (0.1)	18 (1.7)	116 (11.1)
WT vs. non-WT (CLSI)	750 (71.7%)								296 (28.3%)			
WT vs. non-WT (EUCAST)	750 (71.7%)								296 (28.3%)			
**Drug**	**MIC (mg/L)**											
	**≤0.008**	**0.015**	**0.03**	**0.06**	**0.12**	**0.25**	**0.5**	**1**	**2**	**4**	**8**	**>8**
Caspofungin, n (%)	0 (0)	12 (1.1)	213 (20.4)	425 (40.6)	287 (27.4)	80 (7.6)	9 (0.9)	6 (0.6)	4 (0.4)	0 (0)	4 (0.4)	6 (0.6)
S vs. I vs. R (CLSI)	937 (89.6%)					80 (7.6%)	29 (2.8%)					
EUCAST	NA											
**Drug**	**MIC (mg/L)**											
	**≤0.008**	**0.015**	**0.03**	**0.06**	**0.12**	**0.25**	**0.5**	**1**	**2**	**4**	**8**	**> 8**
Micafungin, n (%)	209 (20.0)	734 (70.2)	63 (6.0)	12 (1.1)	8 (0.8)	8 (0.8)	1 (0.1)	2 (0.2)	4 (0.4)	4 (0.4)	1 (0.1)	0
S vs. I vs. R (CLSI)	1018 (97.3%)				8 (0.8%)	20 (1.9%)						
S vs. R (EUCAST)	1006 (96.2%)			40 (3.8%)								
**Drug**	**MIC (mg/L)**											
	**≤0.015**		**0.03**		**0.06**		**0.12**	**0.25**	**0.5**		**1**	**2**
Anidulafungin, n (%)	203 (19.4)		507 (48.5)		212 (20.3)		91 (8.7)	12 (1.1)	6 (0.6)		7 (0.7)	8 (0.8)
S vs. I vs. R (CLSI)	1013 (96.8%)							12 (1.1%)	21 (2.0%)			
S vs. R (EUCAST)	922 (88.1%)						124 (11.9%)					
**Drug**	**MIC (mg/L)**											
	**≤0.012**		**≤0.12**		**0.25**		**0.5**	**1**		**2**		**4**
Amphotericin, n (%)	1 (0.1)		21 (2.0)		127 (12.1)		494 (47.2)	390 (37.3)		13 (1.2)		0 (0%)
WT vs. non-WT (CLSI)	1046 (100%)											0 (0%)
S vs. R (EUCAST)	1033 (98.8%)									13 (1.2%)		
**Drug**	**MIC (mg/L)**											
	**≤0.06**	**0.12**	**0.25**	**1**	**2**		**4**	**8**	**16**	**32**	**64**	**>64**
5-flucytosine, n (%)	1011 (96.7)	11 (1.1)	4 (0.4)	5 (0.5)	10 (1.0)		3 (0.3)	1 (0.1)	0 (0)	0 (0)	1 (0.1)	0 (0)

Abbreviations: CLSI = Clinical and Laboratory Standards Institute; EUCAST = European Committee on Antimicrobial Susceptibility Testing; S = susceptible; SDD = susceptible dose-dependent; I = intermediate; MIC = minimal inhibitory concentration; mg/L = microgram per liter; n = number; NA = not applicable; R = resistant; and WT = wild type.

**Table 3:** Fluconazole susceptibility based on source.

Intra-Abdominal (n = 329)												
Drug	MIC (mg/L)											
	≤0.12	0.5	1	2	4	8	16	32	64	128	256	>256
Fluconazole, n (%)	1 (0.3)	0 (0.0)	2 (0.6)	14 (4.3)	51 (15.5)	103 (31.3)	104 (31.6)	18 (5.5)	8 (2.4)	12 (3.6)	9 (2.7)	7 (2.1)
SDD vs. R (CLSI)	293 (89.1%)								36 (10.9%)			
I vs. R (EUCAST)	275 (83.6%)							54 (16.4%)				
**Blood (n = 247)**												
**Drug**	**MIC (mg/L)**											
	**≤0.12**	**0.5**	**1**	**2**	**4**	**8**	**16**	**32**	**64**	**128**	**256**	**>256**
Fluconazole, n (%)	0 (0.0)	0 (0.0)	1 (0.4)	4 (1.6)	25 (10.1)	80 (32.4)	84 (34.0)	13 (5.3)	7 (2.8)	12 (4.9)	15 (6.1)	6 (2.4)
SDD vs. R (CLSI)	207 (83.8%)								40 (16.2%)			
I vs. R (EUCAST)	194 (78.5%)							53 (21.5%)				
**Urinary (n = 196)**												
**Drug**	**MIC (mg/L)**											
	**≤0.12**	**0.5**	**1**	**2**	**4**	**8**	**16**	**32**	**64**	**128**	**256**	**>256**
Fluconazole, n (%)	0 (0.0)	1 (0.5)	3 (1.5)	11 (5.6)	17 (8.7)	52 (26.5)	49 (25.0)	14 (7.1)	8 (4.1)	21 (10.7)	14 (7.1)	6 (3.1)
SDD vs. R (CLSI)	147 (75.0%)								49 (25.0%)			
I vs. R (EUCAST)	133 (67.9%)							63 (32.1%)				
**Skin and Mucosal (n = 127)**												
**Drug**	**MIC (mg/L)**											
	**≤0.12**	**0.5**	**1**	**2**	**4**	**8**	**16**	**32**	**64**	**128**	**256**	**>256**
Fluconazole, n (%)	0 (0.0)	0 (0.0)	2 (1.6)	2 (1.6)	8 (6.3)	35 (27.6)	31 (24.4)	12 (9.4)	12 (9.4)	14 (11.0)	6 (4.7)	5 (3.9)
SDD vs. R (CLSI)	90 (70.9%)								37 (29.1%)			
I vs. R (EUCAST)	78 (61.4%)							49 (38.6%)				
Other * (n = 147)												
**Drug**	**MIC (mg/L)**											
	**≤0.12**	**0.5**	**1**	**2**	**4**	**8**	**16**	**32**	**64**	**128**	**256**	**>256**
Fluconazole, n (%)	0 (0.0)	0 (0.0)	2 (1.4)	4 (2.7)	17 (11.6)	41 (27.9)	46 (31.3)	12 (8.2)	9 (6.1)	9 (6.1)	3 (2.0)	4 (2.7)
SDD vs. R (CLSI)	122 (83.0%)								25 (17.0%)			
I vs. R (EUCAST)	110 (74.8%)							37 (25.2%)				

* Other sources included bone and joint tissue/fluid (81), respiratory specimens (49), and tissue from the mediastinum and vessels (17). Abbreviations: CLSI = Clinical and Laboratory Standards Institute; EUCAST = European Committee on Antimicrobial Susceptibility Testing; S = susceptible; SDD = susceptible dose-dependent; I = intermediate; MIC = minimal inhibitory concentration; mg/L = microgram per litre; n = number; NA = not applicable; R = resistant; and WT = wild type.

## Data Availability

The data presented in this study are available on request from the corresponding author. The data are not publicly available due to privacy.

## Appendix A

**Table A1:** Cross-resistance/non-wild type for all isolates.

	Fluconazole Resistance Based on CLSI Breakpoints/ECVs (n = 187)	Fluconazole Resistance Based on EUCAST Breakpoints/ECOFFs (n = 256)
Itraconazole non-WT	97 (51.9%)	104 (40.6%)
Voriconazole non-WT	187 (100%)	146 (57.0%)
Posaconazole non-WT	184 (98.4%)	225 (87.9%)
Caspofungin cross-resistance	10 (5.3%)	N/A
Micafungin cross-resistance	8 (4.3%)	17 (6.6%)
Anidulafungin cross-resistance	8 (4.3%)	47 (18.4%)
Amphotericin cross-resistance /non-WT	0 (0%)	6 (2.3%)

Abbreviations: CLSI = Clinical and Laboratory Standards Institute; ECV and ECOFF = epidemiologic cutoff value; EUCAST = European Committee on Antimicrobial Susceptibility Testing; N/A = not applicable; and WT= wild type.

**Table A2:** Cross-resistance/non-wild type for blood isolates.

	Fluconazole Resistance Based on CLSI Breakpoints/ECVs (n = 40)	Fluconazole Resistance Based on EUCAST Breakpoints/ECOFFs (n = 53)
Itraconazole non-WT	22 (55.0%)	24 (45.3%)
Voriconazole non-WT	34 (85.0%)	34 (64.2%)
Posaconazole non-WT	39 (97.5%)	45 (84.9%)
Caspofungin cross-resistance	2 (5.0%)	N/A
Micafungin cross-resistance	1 (2.5%)	4 (7.5%)
Anidulafungin cross-resistance	2 (5.0%)	12 (22.6%)
Amphotericin cross-resistance/non-WT	0 (0.0%)	1 (1.9%)

Abbreviations: CLSI = Clinical and Laboratory Standards Institute; ECV and ECOFF = epidemiologic cutoff value; EUCAST = European Committee on Antimicrobial Susceptibility Testing; N/A = not applicable; and WT= wild type.
